# Cancer-Specific Outcomes in the Elderly with Triple-Negative Breast Cancer: A Systematic Review

**DOI:** 10.3390/curroncol28040215

**Published:** 2021-06-24

**Authors:** Jenny Yoon, Gregory Knapp, May Lynn Quan, Antoine Bouchard-Fortier

**Affiliations:** 1 Department of General Surgery, University of Calgary, Calgary, AB T2N 2T9, Canada; maylynn.quan@albertahealthservices.ca (M.L.Q.); antoine.bouchard-fortier@albertahealthservices.ca (A.B.-F.); 2 Division of General Surgery, Department of Surgery, Dalhousie University, Halifax, NS B3H 2Y9, Canada; knappg@pm.me

**Keywords:** septuagenarians, octogenarians, elderly, TNBC, triple-negative breast cancer, cancer-specific outcome, survival, treatment

## Abstract

Triple-negative breast cancer (TNBC) is more common among young women, although it frequently presents in older patients. Despite an aging population, there remains a paucity of data on the treatment of TNBC in elderly women. We conducted a systematic review of the peer-reviewed and unpublished literature that captures the management and breast-cancer-specific survival (BCSS) of women ≥70 years old with TNBC. Out of 739 papers, five studies met our inclusion criteria. In total, 2037 patients with TNBC treated between 1973 and 2014 were captured in the analysis. Women ≥70 years old were less likely to undergo surgical resection compared to those <70 (92.8% vs. 94.6%, *p* = 0.002). Adjuvant therapy, including radiation and chemotherapy, was also less likely to be utilized in women ≥70 years of age. These treatment differences were associated with more than a doubling of cancer-specific mortality in the elderly cohort (5.9% vs. 2.7% in ≤70 years old, *p* < 0.0001). Two of the five studies showed improved BCSS with adjuvant treatment while others showed no difference. Our systemic review questions the appropriateness of therapeutic de-escalation in this cohort and highlights the significant gap in our understanding of the optimal management for elderly patients with TNBC. Until more data are available, multidisciplinary treatment decision-making should carefully balance the available clinical evidence as well as the patient’s predicted life expectancy and goals-of-care preferences.

## 1. Introduction

Over 250,000 women are diagnosed annually with breast cancer (BC) in the United States [1]. Triple-negative breast cancer (TNBC), which lacks estrogen receptor (ER), progesterone receptor (PR) and human epidermal growth factor receptor 2 (HER2) expression, is associated with a lower disease-free and overall survival compared to the other subtypes [2]. TNBC is associated with a young median age of diagnosis of 54 compared to 60 years for other breast cancer subtypes. Although the odds of having TNBC in women <40 years old is 1.53 times higher than women >60 years old, 15–18% of elderly patients will also be diagnosed with this more aggressive subtype of BC [3,4].

The number of older women affected by breast cancer is projected to increase over the coming decades as the population in North America ages (21.5% ≥65 years old by 2050) [5]. Independent of subtype, a growing body of literature suggests that increasing age is inversely related to the receipt of curative intent surgery and adjuvant therapy (e.g., chemotherapy) [6]. Unfortunately, there are limited data on the efficacy of treatment in patients ≥70 years of age with TNBC with which to make evidenced-based recommendations [7]. To address this shortcoming, we conducted a systematic review of the available data on treatment and breast-cancer-specific outcomes for elderly women with TNBC.

## 2. Methods

### 2.1. Literature Search Method

For this systematic review, we followed the Preferred Reporting Items for Systematic Reviews and Meta-Analyses (PRISMA). We searched for relevant articles in PubMed, Medline, Embase, Cochrane Central Register of Controlled Trials, Web of Science, Scopus and Google Scholar from 1974 to December 2019. This search was supplemented by a manual evaluation of the reference lists of the included studies. Search terms included “triple negative breast cancer”, “triple negative breast neoplasm” or “TNBC” AND “septuagenarian”, “octogenarian”, “nonagenarian”, “centenarian” or “elderly” AND “disease free survival”, “disease specific survival”, “progression free survival” or “cancer specific survival”. 

### 2.2. Selection Criteria

Due to competing mortality risks in the elderly, breast-cancer-specific survival (BCSS) rather than overall survival (OS) was selected as the most appropriate survival outcome. The primary outcome was BCSS. English studies that presented subtype-specific data (i.e., TNBC) for women ≥70 and included treatment details and cancer-specific outcomes were considered for inclusion. Our review included studies that defined elderly with various age thresholds (i.e., ≥65, 70 or 75 years old) as women ≥70 years old were captured in these age groups. Patients with stage IV disease or with incomplete data were excluded from analysis. Eligibility was determined by review of study abstracts. The full texts of articles that remained after abstract screening were reviewed for eligibility for inclusion in qualitative analysis. Two reviewers (JY, SL) independently performed screening of studies for eligibility and data extraction. Discrepancies were resolved by a third investigator (GK). 

### 2.3. Quality Assessment

The Risk of Bias in Non-Randomized Studies of Interventions (ROBINS-I) assessment tool was used to assess for biases due to confounding, selection of participants, classification of interventions, missing data, measurement in outcomes and selection of reported results (Table 1). A moderate level of bias in the selection of reported results was due to multiple measurements within the outcome domain and due to the inclusion of unadjusted hazard ratios. A serious risk of bias due to confounding arose in one study due to the unknown status of the receipt of adjuvant therapy. A moderate level of bias in the selection of participants was found in all studies due to the absence of information on patient comorbidities. 

### 2.4. Data Extraction

The following information was extracted from each study: authors’ names, year of publication, country, study type, total number of patients, patient and tumor characteristics, treatment regimens and BCSS. Breast-cancer specific survival was defined as death attributable to the primary breast cancer diagnosis. Data were extracted independently by two authors for data validation. 

## 3. Results 

### 3.1. Included Studies

**Figure 1:**
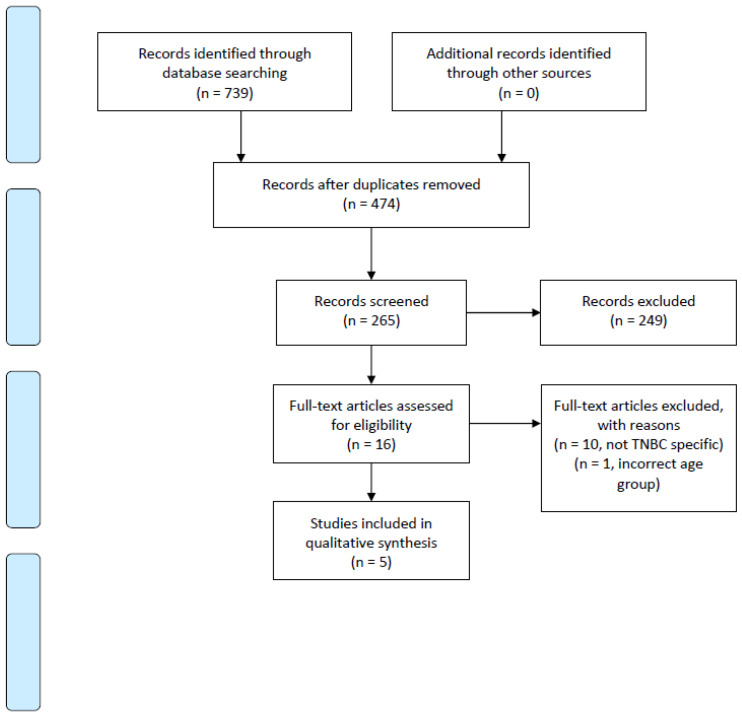
PRISMA 2009 Flow Diagram.

In total, 739 papers were initially identified. After abstract and full-text screening, five studies met a-priori inclusion criteria (Figure 1). A failure to include women ≥70 and a lack of BCSS data were the primary reasons for study exclusion. The included studies were all retrospective, observational studies from the United States, Asia and United Kingdom (Table 2). Three out of the five studies compared women <70 years to women ≥70 years old. The remaining studies included women ≥65 and ≥75 years old. The studies only included patients with TNBC and reported patient and tumor characteristics, such as race, ethnicity, TNM stage and histological grade. Four out of five studies included patients with stage I-III disease, while the remaining study included stages I to II only.

One study reported a relative survival ratio to approximate BCSS by calculating the ratio of overall survival with TNBC to a cohort of women without breast cancer, matched for age, country and year of treatment. Additional outcomes reported included locoregional recurrence, metastatic free and OS. 

### 3.2. Clinicopathologic Features of TNBC Tumor in the Elderly

In total, 2037 patients with TNBC treated between 1973 and 2014 were identified and their data used in this analysis. Forty-five percent of the patients were stage I, 38% stage II and 15% stage III [9,10,12]. Compared to patients <70 years old, patients ≥70 had smaller tumor size, lower number of lymph node metastasis, histological grade and TNM stage [10,12]. Only one study found that those ≥70 years old were more likely to have tumors ≥2 cm (*p =* 0.002) [11]. 

### 3.3. Zhu et al.

Zhu et al., 2015 examined the use of surgery and radiotherapy for women with TNBC <70 years old and women ≥70 years old from 2010 to 2011 [12]. In this study, women ≥70 years of age were less likely to undergo surgical resection compared to those <70 years old (92.8% vs. 94.6%, *p =* 0.002). When the authors controlled for stage of disease, resection rates were comparable between age cohorts for early-stage disease (i.e., stage I and II). However, for patients with stage III disease, women ≥70 years old were significantly less likely to receive surgical excision (85.8% vs. 90.9%, *p =* 0.004). Those treated with surgical excision saw significant improvement in (HR 0.250, 95% CI, 0.186 to 0.337, *p <* 0.001). 

Decreased use of surgical resection and its impact on BCSS in older women was similarly found in the use of adjuvant radiotherapy. Women ≥70 years were less likely to receive adjuvant radiation compared to women <70 years old (61.2% (749/1224) vs. 69.9% (3260/4661), *p <* 0.001). Patients with more advanced disease (i.e., stage II–III) were less likely to receive radiotherapy compared to those with stage I disease (*p <* 0.001). Receiving radiation was associated with improved BCSS (HR 0.504, 95% CI, 0.390 to 0.651, *p <* 0.001). 

The decreased likelihood of older women ≥70 years old receiving both surgical resection and postoperative radiation compared to those <70 years was associated with more than a doubling of cancer-specific mortality in the elderly cohort (5.9% vs. 2.7% in ≤70, *p <* 0.0001). 

### 3.4. Bhoo-Pathy et al.

Bhoo-Pathy et al., 2015 studied the survival benefit of adjuvant radiation after surgical resection in women ≥65 years old who were diagnosed with TNBC from 2006 to 2011 [8]. When comparing the type of surgical resection, the authors found that older women were more likely have mastectomy as opposed to BCS. The median age of patients who underwent BCS was 49 years old and the median age of patients who underwent mastectomy was 59 years old. In regard to radiation use, their findings were similar to Zhu et al., 2015. The authors found that the older age group was less likely to receive radiation. Moreover, 34.9% of women ≥65 years old received postoperative radiation compared to 67.8% of women <65 years old. To approximate the impact of adjuvant radiation on BCSS, the authors used a relative survival ratio. The relative survival ratio was calculated by comparing OS in TNBC patients to the OS of the general female population controlled for age, calendar year and country of residency. This analysis showed that post-mastectomy radiotherapy did not significantly improve 5-year relative survival compared to mastectomy alone (HR 1.34, CI 0.67–2.68, *p* > 0.05). 

### 3.5. Kaplan et al.

In addition to surgery and radiation, use of chemotherapy was examined in a study by Kaplan et al., 2017 [9]. The authors compared treatment modalities for women ≥75 years old with stage I–III TNBC to women <75 years old from 1990 to 2014. They found that older women were more likely to have a mastectomy as opposed to BCS (41% vs. 36%, *p =* 0.009). In contrast to the two previous studies mentioned above, the receipt of post-surgery radiation did not differ between patients <75 and ≥75 years old (76% vs. 79%, *p =* 0.821). Regarding the receipt of chemotherapy, the authors found that 40% (23/59) of women ≥75 received chemotherapy compared to 76% (76/100) in those <75 years of age (*p <* 0.001). More specifically, of the 23 patients ≥75 years, 17 (74%) received adjuvant chemotherapy and six (26%) received neoadjuvant chemotherapy. In the cohort of patients <75 years, 17 (22%) of the 76 received neoadjuvant chemotherapy and 59 (78%) received adjuvant chemotherapy. Elderly patients with TNBC were also less likely to complete the full course of treatment (74% vs. 84%, *p =* 0.001). Kaplan et al., 2017 revealed a multidisciplinary treatment combination of treatment modality in TNBC patients ≥75 years old [9]. Older women were significantly less likely to receive all forms of treatment (surgery, chemotherapy and radiation) compared to those <75 years old (29% vs. 62%, *p <* 0.001). The older age group was also less likely to receive surgery in addition to chemotherapy (10% vs. 14%, *p <* 0.001). This trend was reversed for the management of BC with surgery plus radiation or with surgical resection alone. Older women were more likely to receive surgery and radiation (48% vs. 18%, *p <* 0.001) and surgery alone (14% vs. 6%, *p <* 0.001). This pattern suggested that treatment combinations that included chemotherapy were less likely to be used in the elderly population. Overall, the use rate of different combinations of treatments was not associated with a difference in five-year BCSS (90% < 75 vs. 83% ≥ 75, *p =* 0.322). 

### 3.6. Kozak et al.

Kozak et al., 2019 compared the treatment of women ≥70 years old with stage I–III TNBC from 2010 and 2014 to women <70 years old [10]. Comparable rates of mastectomy and BCS were observed between the two age groups: 50.2% of women ≥70 years old underwent mastectomy and 49.9% of women <70 years old had mastectomy (*p =* 0.74). As for adjuvant therapy, the authors found that older women were less likely to receive radiotherapy and chemotherapy: 46.3% (1954/4221) of women ≥70 years old received radiotherapy compared to 53.6% (8259/15411, *p <* 0.001) of those <70 years old. An even greater difference was found with chemotherapy as only 42.2% (1780/4221) of those ≥70 years received chemotherapy compared to 83.1% (12811/15411) in those <70 (*p <* 0.0001). The authors found that the lower use of adjuvant therapy was associated with a relative increase in cancer-specific mortality by 25% in the elderly population. The three-year mortality rate for patients ≥70 years was 12.8% compared to 10.2% in patients <70 years (*p <* 0.0001). 

### 3.7. Syed et al.

Syed et al., 2014 compared the use and survival benefit of chemotherapy between women ≥70 years old and those <70 years old diagnosed with stage I–II TNBC from 1973 to 2010 [11]. The authors reported that 0% (0/127) patients ≥70 years old received adjuvant chemotherapy compared to 47% (150/319) in those <70 years old. Despite none of the older women receiving chemotherapy, they did not find a statistically significant difference in five-year BCSS between patients <70 and ≥70 years (73% vs. 79%, *p =* 0.39). In addition, the local recurrence-free survival, regional recurrence survival and metastasis-free survival also did not differ significantly (*p* > 0.05). 

## 4. Discussion

Triple-negative breast cancer is associated with worse stage-matched outcomes and a younger median age than other molecular subtypes of breast cancer. As a result, a growing body of evidence supports a subtype-specific, aggressive approach to multidisciplinary management. However, TNBC also occurs in older individuals, where shorter life expectancy and competing comorbidities pose a unique challenge. This is the first systematic review of the management of TNBC in women ≥70 years of age. The review highlights the absence of prospective data, the relative de-escalation of adjuvant therapy based on chronological age and the high breast-cancer-specific mortality among the elderly with TNBC. 

There was a striking absence of literature examining the management of TNBC in patients ≥70 years of age. Despite the paucity of data, our study demonstrated that elderly patients with TNBC were less likely to receive adjuvant therapy, including both chemotherapy and radiotherapy. A chronological age threshold of ≥70 and ≥80 was independently associated with the decreased use of radiotherapy in two independent analyses [13,14]. A lower rate of radiotherapy was also influenced by a higher number of comorbidities, lower income and receipt of chemotherapy. Margin-positive resection and disease >2 cm were also associated with a lower likelihood of adjuvant radiotherapy, which may suggest a more palliative approach in these patients [13,14]. One study found that despite being offered radiotherapy, elderly patients were also more likely to refuse treatment [12]. Elderly patients with TNBC were also less likely to receive chemotherapy. A major factor for the omission of chemotherapy included the concern of tolerability in a population with decreased functional reserve [15,16,17]. Indeed, <40% of patients ≥75 years of age are referred to medical oncology for consideration of chemotherapy, compared to 76% for patients <75 (*p <* 0.001) [9].

Decreased use of adjuvant radiotherapy and chemotherapy in TNBC patients has been shown to be associated with lower rates of OS. However, the benefits seen in survival may be attributed to the selection of healthier individuals. Radiation was more likely to be omitted in elderly patients with a higher number of comorbidities [13,14,18,19,20]. In our study, we examined BCSS as opposed to OS due to the competing risk of mortality from associated comorbidities and age. The increasing number of comorbidities such as heart disease, renal failure, liver disease and stroke that are also present in the elderly with breast cancer are associated with decreased survival. These concomitant health conditions other than breast cancer account for a greater proportion of deaths in those ≥75 years old [21]. BCSS in the elderly as the primary outcome would provide a more meaningful effect in breast cancer treatment as opposed to OS, which may be more influenced by comorbidities. 

Of the five studies that reported BCSS, only two studies demonstrated a cancer-specific survival advantage with receipt of adjuvant therapy. This suggests that possible biological differences may exist between TNBC that presents in elderly women vs. younger women. TNBC in women ≥70 years of age is associated with a lower expression of Ki67 (48.0% vs. 87.7%, *p <* 0.001) and p53 (44.6% vs. 55.6%, *p =* 0.02), and higher expression of Bcl2 (79.3% vs. 43.5%, *p <* 0.001) than patients <70 [11]. These findings suggest that even though elderly patients are less likely to receive adjuvant treatment, perhaps the effect on BCSS may be less pronounced than in younger patients due to the less aggressive form of TNBC in the elderly. The level of expression of Ki67, p53 and Bcl2 could be used to select elderly TNBC patients who may derive the most benefit from adjuvant therapy. 

There are several limitations of this study. Primarily, this review is limited by the small number of included studies and the retrospective, observational nature of the data. The specific radiation and chemotherapy regimens were not reported in some studies. Finally, most studies did not include a detailed analysis of the differences in baseline characteristics (i.e., sociodemographic, comorbidities) between elderly patients who received adjuvant therapy vs. those who did not receive additional treatment. Biological age alone does not dictate an individual’s level of fitness or ability to tolerate adjuvant therapy. Studies have shown that elderly patients in good health may be able to tolerate standard chemotherapy regimens as well as younger patients [22]. Availability of data on comorbidities and frailty, defined as state of vulnerability to poor resolution of homeostasis following a stress due to cumulative decline in multiple physiological systems over a lifespan, would have provided more insight into the differences in treatment received and BCSS in our study as opposed to age alone [23]. 

## 5. Conclusions

Our study demonstrates a significant gap in our understanding of the optimal treatment for elderly patients with TNBC. Exploration of factors such as patient comorbidity and preference, as well as physician bias, leading to the de-escalation of TNBC treatment in the elderly is required. Studies demonstrating a cancer-specific survival benefit with treatment of elderly patients may be due to the selection of healthier individuals. More prospective data are needed to tailor treatment to both the biological age of the patient and the biology of the breast cancer. Until more data are available, treatment decision-making should carefully balance the available clinical evidence from younger cohorts as well as the patient’s predicted life expectancy and goals-of-care preferences. This should be undertaken in the context of a multidisciplinary review. Absent from historical trials, there is a paucity of prospective data with which to guide current management. As the proportion of patients >70 years increases in society, a greater focus is needed on generating data specific to this subgroup of patients in a manner similar to our approach to breast cancer in the very young. 

## Figures and Tables

**Table 1:** The Risk of Bias in Non-Randomized Studies of Interventions (ROBINS-I) assessment tool for included studies.

Author, Year	Bias Due to Confounding	Bias in Selection of Participants	Bias in Classification of Interventions	Bias Due to Missing Data	Bias in Measurement of Outcomes	Bias in Selection of Reported Result
Bhoo-Pathy, 2015 [8]	Low	Moderate	Low	Low	Low	Low
Kaplan, 2017 [9]	Low	Moderate	Low	Low	Low	Moderate
Kozak, 2019 [10]	Low	Moderate	Low	Low	Low	Moderate
Syed, 2014 [11]	Low	Moderate	Low	Low	Low	Moderate
Zhu, 2015 [12]	Serious	Moderate	Low	Low	Low	Moderate

**Table 2:** Study characteristic table of included studies.

Study Characteristics						
Author, Year, Country	Data Source	Study Period	Patients (n), Age Range	Patient and Tumor Characteristics	Treatment Findings	Breast-Cancer-Specific Survival (BCSSS) Outcomes
Bhoo-Pathy, 2015, Asia [8]	5 hospital-based cancer registries	2016–2011	205, 65–96	Ethnicity, T stage, N stage, tumor grade, lymphovascular invasion	Less radiation use ≥65 vs. >65 (34.9% vs.67.8%)	No survival advantage with radiation in ≥65 (HR 1.34, CI 0.67–2.68, *p* > 0.05)
Kaplan, 2017, USA [9]	Institution-specific breast cancer registry data base	1990–2014	59, 75–93	Race, stage, histologic grade, nuclear grade, mean tumor size, N stage	No difference in radiation use ≥75 vs. <75 (79% vs. 75%, *p* = 0.821) Less use of chemotherapy ≥75 vs. <75 (40% vs. 76%, *p* < 0.001) Less use of surgery, radiation and chemotherapy in ≥75 vs. <75 75 (29% vs. 62%, *p <* 0.001)	Less use of surgery, radiation and chemotherapy was not associated with a difference in 5-year BCSS (90% <75 vs. 83% ≥75, *p =* 0.322)
Kozak, 2019, USA [10]	SEER	2010–2014	422, 70–100	Race, region, grade, stage	Less use of radiation in ≥70 vs. <70 (46.3% vs. 53.6% <0.0001) Less use of chemotherapy ≥70 vs. <70 (42.2% vs. 83.1% *p <* 0.0001)	Decreased use of radiation and chemotherapy in ≥70 vs. <70 was associated with a relative increase in cancer-specific mortality by 25% and increased breast cancer mortality rate (12.8% vs. 10.2%, *p <* 0.0001).
Syed, 2014, UK [11]	Prospective single institution	1973–2010	127, 70–91	Histological type, size, axillary lymph node status, grade	Less use of chemotherapy ≥70 vs. >70 (0% vs. 47%)	No difference in BCSS ≥70 vs. <70 (73% vs. 79%, *p =* 0.39)
Zhu, 2015, USA [12]	SEER	2010–2011	1224, 70–100	TN, stage, grade	Less use of surgery in ≥70 vs. <70 (92.8% vs. 94.6%, *p =* 0.002) Less use of RT in ≥70 vs. <70 (69.9% vs. 61.2%, *p <* 0.001)	Surgery BCSS ^1^ HR 0.250, 95% CI, 0.186 to 0.337, *p <* 0.001 Radiation BCSS HR 0.504, 95% CI, 0,390 to 0.651, *p <* 0.001 Decreased surgery and radiation in ≥70 associated with 5.9% cancer-specific mortality vs. 2.7% in <70 (*p <* 0.0001)

^1^ Breast-cancer-specific survival.

## Data Availability

Data used in this study can be found in the included original studies.
